# Impact of the Korean Medical–Political Conflict on Septic Shock Care in the Emergency Department: A Retrospective Single-Center Clinical Analysis

**DOI:** 10.3390/medicina62081604

**Published:** 2026-08-21

**Authors:** Seongmun Kang, Jae Hwan Kim, Chiwon Ahn, Young Taeck Oh, Sojune Hwang

**Affiliations:** Department of Emergency Medicine, College of Medicine, Chung-Ang University, Seoul 06974, Republic of Korea; 07731@cauhs.or.kr (S.K.); cahn@cau.ac.kr (C.A.); powerfreeze@hanmail.net (Y.T.O.); junee3278@cauhs.or.kr (S.H.)

**Keywords:** septic shock, medical–political conflict, healthcare disruption, healthcare workforce shortage

## Abstract

*Background and Objectives*: The 2024 Korean medical–political conflict caused the mass resignation of trainee physicians, raising concerns about the quality of care for time-critical emergencies. This study aimed to assess the impact of workforce disruptions on the management and outcomes of adults presenting to the emergency department (ED) with septic shock. *Materials and Methods*: This retrospective single-center cohort study was conducted at a tertiary care ED and included adults with septic shock between September 2022 and August 2025. This study compared the pre-conflict period, operating under a resident-supported model, with the conflict period characterized by a specialist-led staffing model. The primary outcome was the all-cause in-hospital mortality. The secondary outcomes included process-related time intervals and clinical course measurements. Multivariate logistic regression was used to identify the independent predictors of mortality. *Results*: Altogether, 343 patients were included (159 pre-conflict, 184 conflict). During the conflict, ED crowding significantly decreased (average concurrent ED patients: 14.9 vs. 7.6, *p* < 0.001). Key diagnostic process metrics, including time to blood gas analysis, blood sampling, blood culture, computed tomography, and total ED length of stay, were significantly shortened. Time-critical therapeutic intervals, such as time to empirical antibiotics, remained unchanged. In-hospital mortality did not differ significantly between the pre- and conflict periods. Multivariate analysis demonstrated that the conflict period was not independently associated with mortality; only initial serum lactate levels and Acute Physiology and Chronic Health Evaluation II scores remained significant predictors. *Conclusions*: The 2024 Korean medical–political conflict was not associated with increased in-hospital mortality among patients with septic shock. Process-related metrics improved, while key therapeutic intervals and overall clinical outcomes remained stable. These findings may reflect reduced ED crowding, organizational adaptation, and continued adherence to established septic shock management practices. Reduced ED volume likely contributed substantially to improvements in diagnostic and throughput-related processes.

## 1. Introduction

Septic shock, a time-critical medical emergency characterized by profound circulatory and metabolic failure, remains a leading cause of mortality in emergency departments (EDs) worldwide. Despite advances in critical care, mortality from septic shock remains high, commonly exceeding 30% across diverse healthcare systems [1,2]. In Republic of Korea, the clinical burden is similarly substantial, with recent studies demonstrating persistently high mortality among patients with septic shock [3,4]. Because patient prognosis hinges directly on time-dependent interventions such as the rapid administration of empirical broad-spectrum antibiotics and aggressive fluid resuscitation, international guidelines consistently emphasize early recognition and prompt protocol-driven care as cornerstones of survival [5,6]. Crucially, executing this complex, time-sensitive workflow requires not only clinical adherence but also immediate availability of sufficient healthcare resources and a well-coordinated team of trained medical personnel [5,7]. Given these high resource and personnel requirements, frontline emergency care systems are vulnerable to external structural disruptions or acute workforce shortages that may directly compromise both the efficiency of care delivery and patient outcomes [8,9].

In February 2024, the South Korean healthcare system encountered an unprecedented structural shock when the government’s announcement of medical school quota expansion triggered a massive medical–political conflict. This conflict led to the mass resignation of more than 90% of junior doctors (interns and residents) nationwide, severely compromising the workforce stability of training hospitals that rely heavily on trainee physicians for frontline emergency care [10]. While administrative emergency countermeasures were deployed to redistribute patient volumes, the sudden and prolonged shortage of medical personnel placed an overwhelming burden on the remaining faculty members [11,12]. Frontline EDs faced potential operational bottlenecks leading to documented prehospital delays, and altered in-hospital patient flows [13,14,15].

Despite the growing discourse regarding the systemic strains imposed by this protracted medical crisis, empirical evidence directly quantifying their consequences on hyperacute clinical pathways such as septic shock remains limited. Because septic shock is a highly lethal condition requiring immediate multidisciplinary intervention, its management may be particularly vulnerable to severe healthcare workforce shortages. It remains unclear whether large-scale workforce disruption and rapid reorganization of emergency department staffing compromise survival, or whether institutional adaptation can preserve timely care despite these constraints.

Therefore, this study aimed to assess the impact of the Korean medical–political conflict on the management and outcomes of adult patients with septic shock in the ED. Using a retrospective single-center design, we analyzed process-related metrics and clinical outcomes, including time to antibiotic administration, ED length of stay, intensive care utilization, and in-hospital mortality, while accounting for the concurrent transition in ED staffing from a resident-supported to a faculty-centered model during the conflict period.

## 2. Materials and Methods

### 2.1. Study Design and Setting

This retrospective observational cohort study was conducted in the ED of a tertiary-care university hospital. The ED functions as a regional referral center, providing 24 h emergency care for a broad spectrum of medical and surgical conditions, including critically ill patients requiring intensive care. The total study period spanned three years, from 1 September 2022 to 31 August 2025. To evaluate the impact of the Korean medical–political conflict, this timeframe was divided into two distinct periods based on the onset of healthcare disruption: the pre-conflict period (control cohort: 1 September 2022 to 19 February 2024) under conventional ED staffing and the conflict period (exposure cohort: 20 February 2024 to 31 August 2025), coinciding with the mass resignation of junior doctors nationwide. Overall ED activity decreased substantially during the conflict period compared with the pre-conflict period, with annual ED visits averaging approximately 38,000 before the conflict and 22,000 during the conflict period. Furthermore, the medical–political conflict resulted in a structural reconfiguration of the institutional ED staffing model. In the pre-conflict period, frontline care was delivered by a team consisting of a board-certified emergency medicine (EM) specialist supervising two residents (one senior and one junior) responsible for initial patient assessment and procedural management. During the conflict period, following the mass resignation of trainee physicians, care was provided exclusively by board-certified EM specialists who independently managed all clinical tasks, order entries, and procedures without resident support. The institutional septic shock management protocol remained unchanged throughout the study period. Although the conflict period extended for approximately 18 months, the specialist-led staffing model was maintained throughout the conflict period without major additional operational restructuring. This study was designed and reported in accordance with the Strengthening the Reporting of Observational Studies in Epidemiology (STROBE) statement for observational studies. The completed STROBE checklist is provided as Supplementary Table S1.

### 2.2. Participants

All adult patients (aged ≥18 years) who presented to the ED during the study period and were diagnosed with septic shock were included. Septic shock was defined according to the Sepsis-3 criteria as suspected or confirmed infection requiring vasopressor support to maintain a mean arterial pressure ≥ 65 mm Hg after adequate fluid resuscitation, along with a serum lactate level > 2 mmol/L. Patients were excluded if they had received initial resuscitative management at another institution prior to transfer, as these prior interventions would confound the establishment of a strict “time zero” and the accurate assessment of our ED’s initial process metrics. Patients were also excluded if they were transferred to another hospital before completion of the initial evaluation in the ED. Duplicate visits, cases with incomplete or missing data, patients with documented do-not-resuscitate status at presentation, and those who left the ED against medical advice were also excluded.

### 2.3. Data Collection

Data were extracted from the institutional electronic medical record system using standardized data collection procedures. Baseline and demographic characteristics included age; sex; date of ED arrival; and relevant comorbidities, including hypertension, diabetes mellitus, chronic lung disease, chronic kidney disease, liver disease, and active malignancy. The clinical variables at presentation included the time from symptom onset to ED arrival, initial vital signs (blood pressure, heart rate, respiratory rate, and body temperature), mental status, quick Sequential Organ Failure Assessment score (qSOFA score), primary infection source, and ED crowding at presentation, defined as the total number of concurrent patients in the ED at the time of arrival. Laboratory and diagnostic variables included initial laboratory findings such as white blood cell count, hemoglobin, platelet count, creatinine, serum lactate, C-reactive protein, and procalcitonin. In addition, timestamps for key diagnostic procedures including blood gas analysis, initial blood sampling, simple radiography, and computed tomography (CT) were recorded. The additional variables included ventilatory support and initial hospital disposition. Disease severity was assessed using Acute Physiology and Chronic Health Evaluation II (APACHE II) scores.

### 2.4. Outcome Measurements

The primary outcome was all-cause in-hospital mortality, which was defined as the final disposition (survival or death) at hospital discharge. Secondary outcomes included process-related intervals and clinical course measurements. Process-related variables were defined as the time interval from ED arrival to critical interventions, including empirical antibiotic administration, blood culture acquisition, and initiation of vasopressor therapy. The clinical course and healthcare utilization outcomes included ED length of stay, intensive care unit (ICU) admission, total hospital length of stay, and duration of vasopressor use.

### 2.5. Statistical Analyses

Descriptive statistical analysis was performed to determine the baseline characteristics. Categorical variables were analyzed using Pearson’s chi-squared and Fisher’s exact tests. Continuous variables were evaluated for normality using the Shapiro–Wilk test. Normally distributed data were expressed as the mean ± standard deviation and analyzed using the independent samples *t*-test. Non-normally distributed variables were presented as medians with interquartile ranges [IQR] and analyzed using the Mann–Whitney U test. Univariate and multivariate logistic regression analyses were conducted to determine the independent relationship between conflict period (pre-conflict vs. conflict) and all-cause in-hospital mortality. Potential confounding covariates were selected a priori based on their clinical relevance and previous studies. These covariates included demographic factors (age and sex), parameters reflecting the initial clinical severity (serum lactate level and APACHE II score), time from symptom onset to ED arrival, number of concurrent ED patients at presentation, and time to empirical antibiotic administration. The conflict period was forced into the multivariate model as the primary exposure variable. All potential baseline confounders were simultaneously entered into the model using the forced entry method to calculate the adjusted odds ratios (aORs) and their corresponding 95% confidence intervals (CIs). To ensure the statistical robustness of the multivariate logistic regression model, the goodness-of-fit was assessed using the Hosmer–Lemeshow test. Additionally, variance inflation factors (VIFs) were calculated to rule out multicollinearity among the predictor variables, with a VIF of less than 5 considered acceptable. All statistical tests were two-tailed, and a *p*-value < 0.05 was considered statistically significant. All statistical analyses were performed using R (version 4.6.0, R Foundation for Statistical Computing, Vienna, Austria).

### 2.6. Ethics Statement

The study protocol was approved by the Institutional Review Board of the Chung-Ang University Hospital in March 2026 (IRB No. 2603-010-19617). The requirement for informed consent was waived owing to the retrospective nature of the study and the use of anonymized clinical data.

## 3. Results

### 3.1. Baseline Characteristics

**Figure 1:**
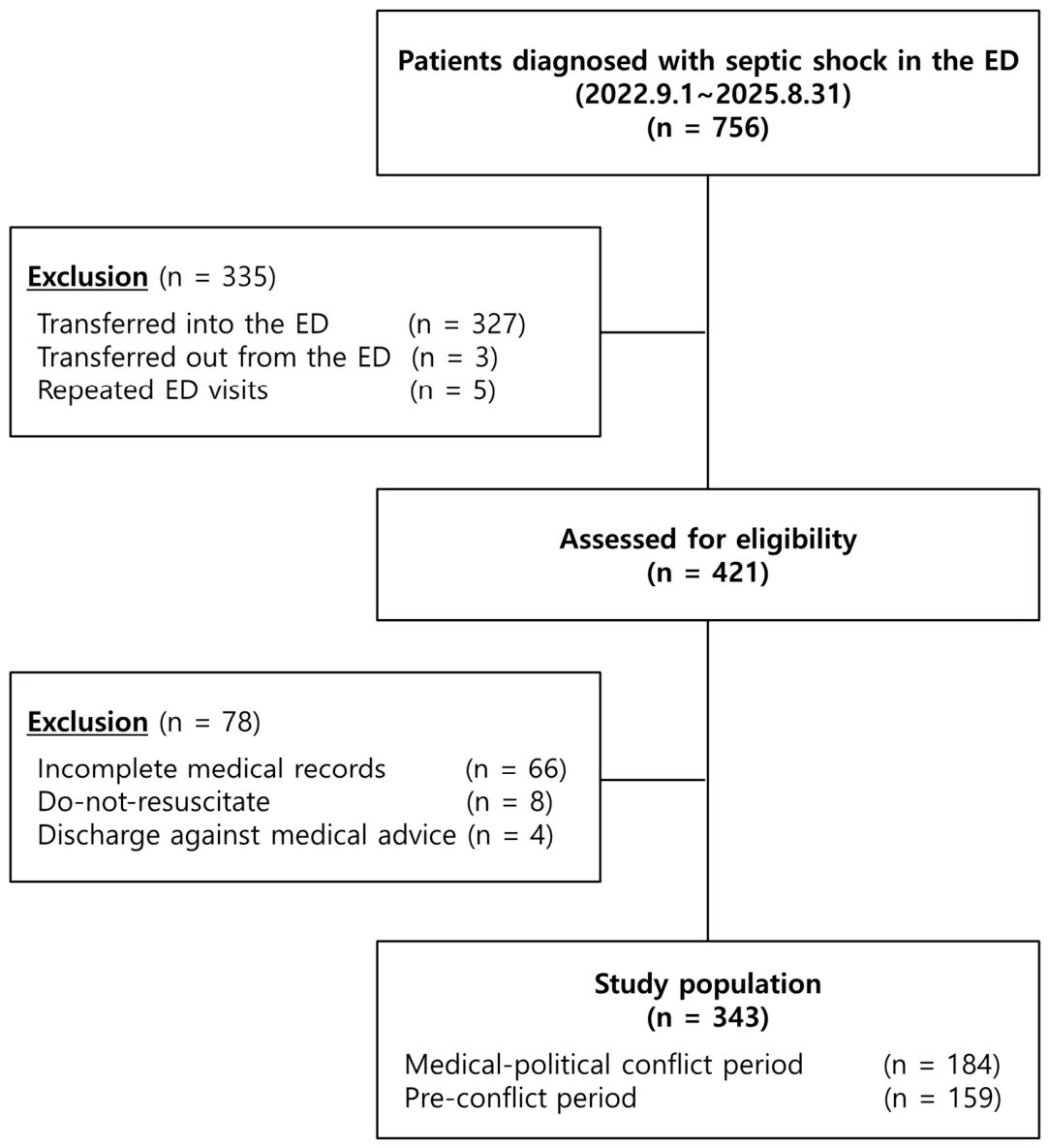
Flow diagram of the study population. ED, emergency department.

Of the 756 patients initially evaluated, 343 with septic shock were included in the final analysis. We excluded 335 patients (327 transferred into the ED, 3 transferred out, and 5 with repeated ED visits), as well as 78 patients with incomplete records, do-not-resuscitate orders, or discharge against medical advice. The cohort comprised 159 patients in the pre-conflict period and 184 patients in the medical–political conflict period (Figure 1). The baseline characteristics were largely comparable between the two periods, except for chronic renal disease, creatinine levels, and hemoglobin levels (Table 1). There were no significant differences in median age (77.0 vs. 79.0 years, *p* = 0.596) or proportion of male patients (49.1% vs. 46.7%, *p* = 0.749) between the pre-conflict and conflict groups. Initial severity markers were also comparable, including the APACHE II score (17.0 [12.0–23.0] vs. 16.0 [11.5–21.5], *p* = 0.332), qSOFA score ≥ 2 (34.0% vs. 39.7%, *p* = 0.441), and serum lactate level (3.9 [2.4–7.0] vs. 3.9 [2.4–6.4] mmol/L, *p* = 0.940). The number of concurrent ED patients was significantly lower during the conflict period (7.6 ± 3.2 vs. 14.9 ± 6.1, *p* < 0.001), and ICU admission was more frequent in the conflict period (77.7% vs. 66.7%, *p* = 0.022).

### 3.2. Process Metrics and Clinical Outcomes

The emergency care process metrics and clinical outcomes for both periods are summarized in Table 2. Compared with the pre-conflict period, the conflict period was associated with a significantly shorter time to blood gas analysis (16.1 [11.3–27.7] vs. 14.3 [9.9–21.2] min, *p* = 0.018), blood sampling (13.8 [9.8–22.0] vs. 12.6 [7.7–17.5] min, *p* = 0.035), blood culture acquisition (30.6 [17.2–67.6] vs. 21.6 [13.1–54.6] min, *p* = 0.011), simple radiography (39.1 [26.3–57.1] vs. 33.0 [20.7–50.1] min, *p* = 0.009), and CT imaging (141.4 [101.1–181.5] vs. 110.6 [86.8–139.4] min, *p* < 0.001). Accordingly, the total ED length of stay was significantly shorter during the conflict period than during the pre-conflict period (3.7 [3.1–4.8] vs. 5.5 [4.4–7.2] h, *p* < 0.001). In contrast, time to antibiotics (84.0 [51.5–128.5] vs. 84.5 [49.0–132.5] min, *p* = 0.614) and time to vasopressor initiation (135.0 [82.5–242.0] vs. 113.5 [65.5–198.0] min, *p* = 0.064) did not differ significantly between the two periods.

In terms of clinical outcomes, no significant differences were observed in the median duration of vasopressor use (2.0 vs. 2.0 d, *p* = 0.322), total hospital length of stay (14.0 vs. 16.0 d, *p* = 0.342), or ICU stay duration (4.0 vs. 4.0 d, *p* = 0.233). Most importantly, the in-hospital mortality rate did not differ significantly between the pre-conflict and conflict periods (28.3% vs. 27.2%, *p* = 0.911).

### 3.3. Factors Associated with In-Hospital Mortality

**Figure 2:**
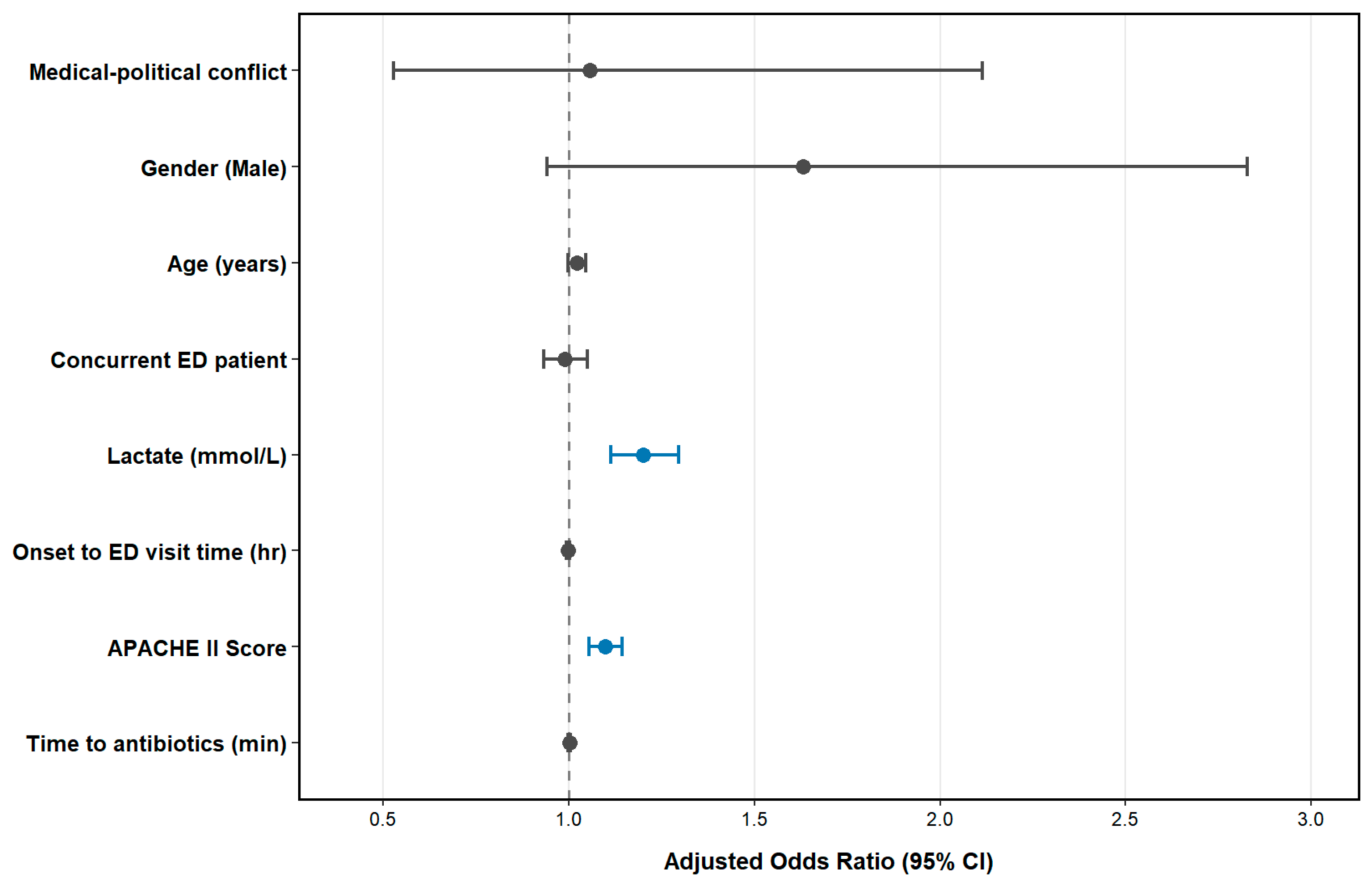
Forest plot of adjusted odds ratios for predictors of in-hospital mortality in patients with septic shock. CI, confidence interval; ED, emergency department; APACHE, Acute Physiology and Chronic Health Evaluation. Blue symbols indicate variables with statistically significant associations (*p* < 0.05), whereas black symbols indicate variables without statistical significance.

Univariate and multivariate logistic regression analyses were performed to identify independent predictors of all-cause in-hospital mortality. In the univariate analysis, higher baseline serum lactate levels (odds ratio (OR), 1.237; 95% CI, 1.155–1.324; *p* < 0.001) and higher initial APACHE II scores (OR, 1.130; 95% CI, 1.088–1.173; *p* < 0.001) were significantly associated with in-hospital mortality, whereas the medical–political conflict period was not (OR, 0.945; 95% CI, 0.589–1.518; *p* = 0.816). In the multivariate logistic regression model, serum lactate levels remained independently associated with in-hospital mortality (aOR, 1.200; 95% CI, 1.112–1.296; *p* < 0.001), as did the APACHE II score (aOR, 1.098; 95% CI, 1.054–1.144; *p* < 0.001). In contrast, the period of medical–political conflict was not significantly associated with in-hospital mortality (aOR, 1.056; 95% CI, 0.527–2.114; *p* = 0.878; Table 3, Figure 2). The multivariate model demonstrated an adequate fit to the data (Hosmer–Lemeshow test, *p* = 0.436), and no significant multicollinearity was observed among the variables (all VIFs < 2.0).

## 4. Discussion

In this retrospective single-center cohort study of adult patients with septic shock, the Korean medical–political conflict was not associated with increased in-hospital mortality despite major changes in emergency department staffing and care delivery processes. During the conflict period, several diagnostic and throughput-related process metrics, including time to blood gas analysis, blood sampling, blood culture acquisition, imaging, and total ED length of stay, were significantly shortened, whereas the time to antibiotic and vasopressor initiation remained unchanged. Multivariate analysis further showed that serum lactate level and APACHE II score, rather than the conflict period itself, were independently associated with in-hospital mortality. Overall, these findings suggest that core septic shock management pathways are preserved during periods of substantial workforce disruption.

The most clinically important finding was that the conflict period was not independently associated with mortality in patients with septic shock. Septic shock is highly time-sensitive, and current guidelines emphasize prompt antimicrobial therapy and rapid hemodynamic resuscitation as key determinants of outcomes [5,6]. In this context, the absence of a mortality difference is notable, particularly because the staffing model shifted from a resident-supported system to a faculty-centered model during the conflict period. Following the withdrawal of trainee physicians, our ED rapidly reorganized staff around board-certified emergency physicians who directly managed all aspects of patient care. Although this transition inherently reduced the overall workforce, the simplified specialist-led structure may have facilitated organizational adaptation through more direct physician involvement. However, the independent contribution of the specialist-led staffing model to workflow improvement could not be determined, because it occurred concurrently with a substantial reduction in ED patient volume.

The reduction in concurrent ED patients during the conflict period is likely a major contributor to the shorter process times. Because ED crowding is known to delay time-sensitive sepsis care, lower congestion can reduce operational bottlenecks, which may explain the shorter times to blood gas analysis, blood sampling and imaging, and the shorter ED length of stay observed in the conflict group [9]. A plausible explanation is that government-led emergency healthcare policies implemented during the 2024 medical crisis redirected patients with low-acuity conditions toward secondary hospitals and community emergency facilities, while tertiary hospitals prioritized the care of critically ill patients. This redistribution likely contributed to the reduction in lower-acuity ED visits observed at tertiary centers [15,16,17]. Although reduced crowding likely contributed to the shorter overall ED length of stay, downstream ICU or general ward bed availability may also have played a role. While hospital-wide bed occupancy was not formally assessed, substantial delays following admission decisions were not considered a prominent feature of our setting. Taken together, these findings highlight the importance of considering ED crowding when interpreting the observed workflow improvements. For our primary outcome of in-hospital mortality, we specifically addressed this confounding through statistical adjustment. By including the number of concurrent ED patients as an a priori covariate in our multivariable model, we observed that the conflict period was not independently associated with mortality. Therefore, while reduced crowding likely facilitated faster workflows, the conflict period was not independently associated with mortality after accounting for this volumetric shift.

However, crowding reduction alone may not fully explain the preservation of core therapeutic windows. The most critical therapeutic intervals, including the time to antibiotic administration and vasopressor initiation, were not significantly different between the periods. Early antibiotic administration has long been recognized as a key component of sepsis and septic shock management. International guidelines continue to emphasize the need for immediate or early antimicrobial treatment in patients with septic shock [5,6]. In our ED, this aspect of care had already been firmly embedded in protocolized sepsis bundles and routine practice before the conflict, which may have rendered antibiotic timing relatively resistant to changes in crowding or staffing. Moreover, antibiotic initiation depends not only on the speed of diagnostic testing but also on clinical decision-making, the selection of appropriate agents, and safety considerations, limiting the extent to which faster workflow alone can shorten this interval. It is also possible that clinicians maintained particular vigilance for this metric during the conflict, because it is widely recognized as a high-priority intervention. Thus, while reduced crowding likely contributed substantially to the accelerated diagnostic workflows, the stability of core therapeutic intervals may reflect the persistence of established sepsis protocols and routine practice across both study periods.

Another notable finding was the higher ICU admission rate during the conflict period despite comparable baseline severity, as reflected by similar APACHE II scores. This may reflect a more proactive triage strategy under the specialist-led model, in which faculty physicians may have favored earlier ICU admission for patients with borderline hemodynamic instability. Such a shift may represent an adaptive safety strategy rather than worsening illness severity. Alternatively, reduced overall hospital activity may have increased ICU bed availability and facilitated ICU admission, particularly for patients with borderline indications for intensive care. Although the higher ICU admission rate may have supported closer monitoring and earlier escalation of care, its contribution to the comparable mortality outcomes remains uncertain because ICU occupancy, bed availability, and detailed post-ED management were not formally assessed.

The absence of a difference in mortality should also be interpreted in light of the multifactorial nature of septic shock outcomes. In-hospital mortality is influenced not only by ED processes but also by baseline comorbidities, initial physiologic severity, infection source, organ dysfunction, response to resuscitation, and subsequent inpatient and ICU management. In our multivariate model, higher serum lactate levels and APACHE II scores were independently associated with mortality, whereas the conflict period was not. Although some baseline characteristics, including chronic kidney disease, initial serum creatinine, and hemoglobin levels, differed between the two groups, our multivariable model was constructed based on an a priori selection of established prognostic factors rather than univariable differences. We utilized the APACHE II score to adjust for overall illness severity; adding individual parameters such as serum creatinine may have introduced redundancy because related aspects of physiologic severity are already represented within the APACHE II score, while inclusion of multiple additional baseline variables could compromise model parsimony and increase the risk of overfitting. Nevertheless, because the primary multivariable model was based on an a priori selection of prognostic factors and the study had a modest sample size, no additional post hoc adjustment analyses were performed. Therefore, residual confounding from these unadjusted baseline differences cannot be completely excluded. Specifically, the significantly lower proportion of patients with chronic kidney disease during the conflict period might have influenced triage decisions. Patients with fewer severe comorbidities may be more readily considered for aggressive care, potentially contributing to the higher ICU admission rate. This pattern underscores the importance of patient-level severity in determining outcomes and may partly explain why differences in ED process measures did not translate into measurable differences in mortality rates.

These findings have implications for healthcare policies and emergency preparedness. Large-scale workforce disruptions are expected to negatively affect the quality of care delivered to critically ill patients. However, our results suggest that the ultimate impact of such disruptions may depend not only on workforce reduction but also on concurrent changes in patient volume, resource availability, and institutional adaptability. Maintenance of established treatment pathways, preservation of access to critical care resources, and rapid organizational restructuring may be particularly important for sustaining quality of care during healthcare crises.

This study had several limitations. First, this was a retrospective single-center study, and unmeasured confounding factors could not be excluded. Furthermore, the exclusion of 327 transferred-in patients may have introduced selection bias, as changes in inter-hospital referral pathways during the conflict could have altered the clinical profile of transferred patients, limiting the generalizability of the findings to patients requiring inter-hospital transfer. Second, because the study was observational, the relative contributions of staffing reorganization, reduced ED crowding, and other institutional adaptations could not be fully ascertained. Third, some important determinants of septic shock outcomes occur after ED management, including ICU-level care, source control, and inpatient complications; these factors were not fully captured in the present analysis. Fourth, although no statistically significant difference in in-hospital mortality was observed between the two periods, the relatively modest sample size may have limited the ability to detect meaningful differences in mortality or treatment delays. Therefore, the possibility of a Type II error cannot be entirely excluded. Nevertheless, the observed mortality rates were highly similar between groups, and the findings should be interpreted within the context of the study’s observational design. Fifth, our primary outcome was all-cause in-hospital mortality, and post-discharge vital status was not systematically available; 28- or 30-day mortality could not be assessed. Given the variability in hospital length of stay, in-hospital mortality may be influenced by differences in discharge practices between periods, and our results should therefore be interpreted with this limitation in mind.

To address these limitations, future research should adopt multicenter, large-scale designs utilizing nationwide registries to improve generalizability and statistical power. Additionally, analyzing comprehensive clinical databases that incorporate detailed post-ED variables—such as the exact timing of source control and specific ICU interventions—is necessary. Finally, comparative observational analyses across diverse healthcare settings with varying degrees of workforce disruption and crowding will be crucial to disentangle the independent effects of specific staffing and operational adaptations.

## 5. Conclusions

The 2024 Korean medical–political conflict was not associated with worse survival outcomes among patients presenting to a tertiary-care ED with septic shock. During the conflict period, several diagnostic and throughput-related process metrics improved, while key therapeutic intervals and overall clinical outcomes remained stable. The potential adverse effects of this healthcare disruption may have been mitigated by reduced ED crowding, organizational adaptation to the changing care environment, and continued adherence to established septic shock management practices. Reduced ED volume likely contributed substantially to the observed improvements in diagnostic and throughput-related processes. Although derived from a single-center observational experience, these findings provide insight into how emergency care systems may maintain critical care delivery during periods of substantial healthcare workforce disruption.

## Figures and Tables

**Table 1:** Baseline characteristics.

Variables	Pre-Conflict Period (N = 159)	Conflict Period (N = 184)	Total (N = 343)	*p* Value
Gender				
Male	78 (49.1%)	86 (46.7%)	164 (47.8%)	0.749
Female	81 (50.9%)	98 (53.3%)	179 (52.2%)	
Age (yr)	77.0 [70.0; 85.0]	79.0 [69.5; 85.0]	78.0 [70.0; 85.0]	0.596
Concurrent ED patient	14.9 ± 6.1	7.6 ± 3.2	11.0 ± 6.0	<0.001
Comorbidities				
Hypertension	99 (62.3%)	111 (60.3%)	210 (61.2%)	0.798
Diabetes	67 (42.1%)	70 (38.0%)	137 (39.9%)	0.508
Chronic lung disease	17 (10.7%)	21 (11.4%)	38 (11.1%)	0.968
Chronic liver disease	15 (9.4%)	11 (6.0%)	26 (7.6%)	0.317
Chronic renal disease	38 (23.9%)	20 (10.9%)	58 (16.9%)	0.002
Malignancy	54 (34.0%)	59 (32.1%)	113 (32.9%)	0.797
Lab data				
WBC (×10^9^/L)	11.0 [6.1; 17.2]	10.9 [6.5; 17.0]	11.0 [6.2; 17.1]	0.962
Hemoglobin (g/dL)	10.8 ± 2.3	11.4 ± 2.5	11.1 ± 2.4	0.039
Platelet (×10^9^/L)	168.0 [114.0; 247.0]	173.0 [111.5; 243.0]	171.0 [113.0; 244.0]	0.989
Creatinine (mg/dL)	1.9 [1.2; 3.2]	1.7 [1.1; 2.4]	1.8 [1.1; 2.8]	0.042
Lactate (mmol/L)	3.9 [2.4; 7.0]	3.9 [2.4; 6.4]	3.9 [2.4; 6.7]	0.940
CRP (mg/L)	136.9 [57.9; 231.9]	107.8 [46.8; 208.1]	122.2 [54.9; 220.3]	0.053
Procalcitonin (ng/mL)	6.3 [1.2; 29.2]	4.6 [0.7; 19.7]	4.8 [0.9; 25.4]	0.156
Infection site				
Respiratory	60 (37.7%)	72 (39.1%)	132 (38.5%)	0.151
Urinary	39 (24.5%)	51 (27.7%)	90 (26.2%)	
Hepatobiliary	26 (16.4%)	19 (10.3%)	45 (13.1%)	
Gastrointestinal	16 (10.1%)	30 (16.3%)	46 (13.4%)	
Bone or soft tissue	8 (5.0%)	8 (4.3%)	16 (4.7%)	
Others or unknown	10 (6.3%)	4 (2.3%)	14 (4.1%)	
Onset to ED visit time (h)	7.9 [1.5; 22.9]	8.7 [2.2; 23.4]	8.3 [1.8; 23.0]	0.255
qSOFA score				
qSOFA ≥ 2, n (%)	54 (34.0%)	73 (39.7%)	127 (37.0%)	0.441
qSOFA ≤ 1, n (%)	105 (66.0%)	111 (60.3%)	216 (63.0)	
APACHE II score	17.0 [12.0; 23.0]	16.0 [11.5; 21.5]	17.0 [12.0; 22.0]	0.332
Ventilation support	42 (26.4%)	53 (28.8%)	95 (27.7%)	0.622
Admission type				0.022
Intensive care unit	106 (66.7%)	143 (77.7%)	249 (72.6%)	
General ward	50 (31.4%)	41 (22.3%)	91 (26.5%)	
Expire in ED	3 (1.9%)	0 (0%)	3 (0.9%)	

Values are shown as the mean ± standard deviation, the median [interquartile range], or frequency (proportion). ED, emergency department; WBC, white blood cell; CRP, C-reactive protein; qSOFA, quick Sequential Organ Failure Assessment; APACHE II, Acute Physiology and Chronic Health Evaluation II. For descriptive analyses, qSOFA scores (range 0–3) were additionally dichotomized using the conventional cut-off of ≥2 versus ≤1 points.

**Table 2:** Process metrics and clinical outcomes.

Variables	Pre-Conflict Period (N = 159)	Conflict Period (N = 184)	Total (N = 343)	*p* Value
**Process metrics**				
Time to BGA (min)	16.1 [11.3; 27.7]	14.3 [9.9; 21.2]	14.9 [10.6; 24.3]	0.018
Time to blood sampling (min)	13.8 [9.8; 22.0]	12.6 [7.7; 17.5]	13.5 [8.9; 19.9]	0.035
Time to blood culture (min)	30.6 [17.2; 67.6]	21.6 [13.1; 54.6]	25.5 [15.6; 60.4]	0.011
Time to antibiotics (min)	84.0 [51.5; 128.5]	84.5 [49.0; 132.5]	84.0 [50.0; 130.0]	0.614
Time to vasopressor (min)	135.0 [82.5; 242.0]	113.5 [65.5; 198.0]	126.0 [70.0; 219.5]	0.064
Time to simple radiography (min)	39.1 [26.3; 57.1]	33.0 [20.7; 50.1]	36.6 [22.6; 52.8]	0.009
Time to CT (n = 311) (min)	141.4 [101.1; 181.5] (n = 139)	110.6 [86.8; 139.4] (n = 172)	121.4 [90.7; 163.7]	<0.001
Total ED length of stay (h)	5.5 [4.4; 7.2]	3.7 [3.1; 4.8]	4.5 [3.4; 5.9]	<0.001
**Clinical outcomes**				
Duration of vasopressor use	2.0 [1.0; 5.0]	2.0 [1.0; 4.0]	2.0 [1.0; 5.0]	0.322
Total hospital length of stay	16.0 [9.0; 29.0]	14.0 [8.0; 26.0]	15.0 [8.5; 28.0]	0.342
ICU staying days (n = 249)	4.0 [2.0; 7.0]	4.0 [2.0; 10.0]	4.0 [2.0; 9.0]	0.233
In-hospital mortality	45 (28.3%)	50 (27.2%)	95 (27.7%)	0.911

Values are shown as the median [interquartile range] or frequency (proportion). BGA, blood gas analysis; CT, computed tomography; ED, emergency department; ICU, intensive care unit.

**Table 3:** Univariable and multivariable logistic regression analysis of in-hospital mortality.

Variable	Univariable OR (95% CI)	*p*-Value	Multivariable aOR (95% CI)	*p*-Value
Medical–political conflict	0.945 (0.589–1.518)	0.816	1.056 (0.527–2.114)	0.878
Gender, male	1.468 (0.913–2.362)	0.113	1.631 (0.941–2.827)	0.081
Age, years	1.015 (0.996–1.035)	0.127	1.021 (0.998–1.045)	0.069
Concurrent ED patient	1.016 (0.977–1.056)	0.436	0.990 (0.933–1.051)	0.735
Lactate (mmol/L)	1.237 (1.155–1.324)	<0.001	1.200 (1.112–1.296)	<0.001
Onset to ED visit time (h)	0.995 (0.990–1.001)	0.081	0.998 (0.993–1.002)	0.326
APACHE II Score	1.130 (1.088–1.173)	<0.001	1.098 (1.054–1.144)	<0.001
Time to antibiotics (min)	1.000 (0.996–1.003)	0.814	1.001 (0.997–1.005)	0.706

ED, emergency department; APACHE II, Acute Physiology and Chronic Health Evaluation II.

## Data Availability

The datasets generated during the current study are available from the corresponding author on reasonable request.

## Supplementary Materials

The following supporting information can be downloaded at: https://www.mdpi.com/article/10.3390/medicina62081604/s1, Table S1. STROBE Statement checklist for observational studies.

## Abbreviations

The following abbreviations are used in this manuscript:

aOR	Adjusted odds ratio
APACHE	Acute Physiology and Chronic Health Evaluation
CI	Confidence interval
CT	Computed tomography
ED	Emergency department
EM	Emergency medicine
ICU	Intensive care unit
IQR	Interquartile range
qSOFA	Quick Sequential Organ Failure Assessment
STROBE	Strengthening the Reporting of Observational Studies in Epidemiology
VIF	Variance inflation factors
