# Supplementary material for: Impact of the Korean Medical–Political Conflict on Septic Shock Care in the Emergency Department: A Retrospective Single-Center Clinical Analysis
Source: Medicina (Kaunas). 2026 Aug 21;62(8):1604. doi: 10.3390/medicina62081604 (PMC13515901; doi:10.3390/medicina62081604)
Supplement: Supplementary file 1 [file medicina-62-01604-s001.zip › medicina-4454634-supplementary.pdf]

Supplementary Table S1. STROBE Statement checklist for observational studies

	Item No.	Recommendation	Page No.	Relevant text from manuscript
**Title and abstract**	1	(*a*) Indicate the study's design with a commonly used term in the title or the abstract	Page 1.	Title: "...A Retrospective Single-Center Clinical Analysis" Abstract: "This retrospective single-center cohort study was conducted..."
		(*b*) Provide in the abstract an informative and balanced summary of what was done and what was found	Page 1.	The abstract provides a structured summary including Background, Methods, Results, and Conclusions.
**Introduction**				
Background/rationale	2	Explain the scientific background and rationale for the investigation being reported	Page 2.	Introduction, paragraphs 1–3
Objectives	3	State specific objectives, including any prespecified hypotheses	Page 2.	Introduction, paragraph 4 "Therefore, this study aimed to assess the impact of the Korean medical–political conflict on the management and outcomes of adult patients with septic shock in the ED."
**Methods**				
Study design	4	Present key elements of study design early in the paper	Page 3.	2.1. Study design and setting "This retrospective observational cohort study was conducted in the ED of a tertiary-care university hospital. "
Setting	5	Describe the setting, locations, and relevant dates, including periods of recruitment, exposure, follow-up, and data collection	Page 3.	2.1. Study design and setting "The total study period spanned three years, from September 1, 2022, to August 31, 2025... divided into two distinct periods based on the onset of healthcare disruption..."

Participants	6	(*a*) *Cohort study*—Give the eligibility criteria, and the sources and methods of selection of participants. Describe methods of follow-up<br>*Case-control study*—Give the eligibility criteria, and the sources and methods of case ascertainment and control selection. Give the rationale for the choice of cases and controls<br>*Cross-sectional study*—Give the eligibility criteria, and the sources and methods of selection of participants	Page 3.	2.2. Participants<br>"All adult patients (aged ≥ 18 years) who presented to the ED during the study period and were diagnosed with septic shock were included... Patients were excluded if they had received initial resuscitative management at another institution... "
		(*b*) *Cohort study*—For matched studies, give matching criteria and number of exposed and unexposed<br>*Case-control study*—For matched studies, give matching criteria and the number of controls per case	N/A	N/A
Variables	7	Clearly define all outcomes, exposures, predictors, potential confounders, and effect modifiers. Give diagnostic criteria, if applicable	Page 3-4.	2.2. Participants<br>"Septic shock was defined according to the Sepsis-3 criteria..."<br>2.4. Outcome Measurements<br>"The primary outcome was all-cause in-hospital mortality..."<br>2.5. Statistical Analyses<br>"Potential confounding covariates were selected a priori... These covariates included demographic factors... and number of concurrent ED patients at presentation..."
Data sources/ measurement	8*	For each variable of interest, give sources of data and details of methods of assessment (measurement). Describe comparability of assessment methods if there is more than one group	Page 3-4.	2.3. Data Collection<br>"Data were extracted from the institutional electronic medical record system using standardized data collection procedures."
Bias	9	Describe any efforts to address potential sources of bias	Page 3-4.	2.2. Participants<br>"Patients were excluded if they had

				received initial resuscitative management at another institution... as these prior interventions would confound the establishment of a strict 'time zero'..."<br>2.5. Statistical Analyses<br>"All potential baseline confounders were simultaneously entered into the model using the forced entry method..."
Study size	10	Explain how the study size was arrived at	Page 3,5.	2.2. Participants<br>"All adult patients (aged ≥ 18 years) who presented to the ED during the study period and were diagnosed with septic shock were included."<br>3.1. Baseline characteristics<br>"Of the 756 patients initially evaluated, 343 with septic shock were included in the final analysis after excluding 335 patients..."

Continued on next page

Quantitative variables	11	Explain how quantitative variables were handled in the analyses. If applicable, describe which groupings were chosen and why	Page 4.	2.5. Statistical Analyses "Continuous variables were evaluated for normality using the Shapiro–Wilk test. Normally distributed data were expressed as the mean ± standard deviation... Non-normally distributed variables were presented as medians with interquartile ranges [IQR]..."
Statistical methods	12	(*a*) Describe all statistical methods, including those used to control for confounding	Page 4.	2.5. Statistical Analyses "Univariate and multivariate logistic regression analyses were conducted... The conflict period was forced into the multivariate model as the primary exposure variable."
		(*b*) Describe any methods used to examine subgroups and interactions	N/A	N/A
		(*c*) Explain how missing data were addressed	Page 5.	3.1. Baseline characteristics "...excluding... 78 patients with incomplete records, do-not-resuscitate orders, or discharge against medical advice." (Cases with missing/incomplete data were excluded upfront).
		(*d*) *Cohort study*—If applicable, explain how loss to follow-up was addressed<br>*Case-control study*—If applicable, explain how matching of cases and controls was addressed<br>*Cross-sectional study*—If applicable, describe analytical methods taking account of sampling strategy	N/A	N/A
		(*e*) Describe any sensitivity analyses	N/A	N/A
**Results**				
Participants	13*	(a) Report numbers of individuals at each stage of study—eg numbers potentially eligible, examined for eligibility, confirmed eligible, included in the study, completing follow-up, and analysed	Page 5.	3.1. Baseline characteristics
		(b) Give reasons for non-participation at each stage	Page 5.	3.1. Baseline characteristics
		(c) Consider use of a flow diagram	Figure 1.	

Descriptive data	14*	(a) Give characteristics of study participants (eg demographic, clinical, social) and information on exposures and potential confounders	Page 5.<br>Page 13-14.	Table 1 & 3.1. Baseline characteristics
		(b) Indicate number of participants with missing data for each variable of interest	Figure 1.	
		(c) *Cohort study*—Summarise follow-up time (eg, average and total amount)	Page 15.	Patients were followed from ED arrival through their entire hospitalisation; follow-up time is reflected by total hospital length of stay and ICU stay duration, which are reported as medians with interquartile ranges in Table 2.
Outcome data	15*	*Cohort study*—Report numbers of outcome events or summary measures over time	Page 5, 15.	Table 2 & 3.2. Process Metrics and Clinical Outcomes
		Case-control study—Report numbers in each exposure category, or summary measures of exposure	N/A	N/A
		Cross-sectional study—Report numbers of outcome events or summary measures	N/A	N/A
Main results	16	(*a*) Give unadjusted estimates and, if applicable, confounder-adjusted estimates and their precision (eg, 95% confidence interval). Make clear which confounders were adjusted for and why they were included	Page 6.<br>Page 16.	Table 3 & 3.3. Factors Associated with In-Hospital Mortality<br>Figure 2.
		(*b*) Report category boundaries when continuous variables were categorized	Page 14.	The category boundaries are reported in the footnote of Table 1.
		(*c*) If relevant, consider translating estimates of relative risk into absolute risk for a meaningful time period	N/A	N/A

Continued on next page

Other analyses	17	Report other analyses done—eg analyses of subgroups and interactions, and sensitivity analyses	N/A	N/A
**Discussion**				
Key results	18	Summarise key results with reference to study objectives	Page 7.	Discussion, paragraph 1
Limitations	19	Discuss limitations of the study, taking into account sources of potential bias or imprecision. Discuss both direction and magnitude of any potential bias	Page 9.	Discussion, paragraph 8
Interpretation	20	Give a cautious overall interpretation of results considering objectives, limitations, multiplicity of analyses, results from similar studies, and other relevant evidence	Page 7-9.	Discussion, paragraphs 2-7
Generalisability	21	Discuss the generalisability (external validity) of the study results	Page 9-10.	"Furthermore, the exclusion of transferred patients may have introduced selection bias and limited the generalizability of the findings." "To address these limitations, future research should adopt multicenter, large-scale designs utilizing nationwide registries to improve generalizability and statistical power. "
**Other information**				
Funding	22	Give the source of funding and the role of the funders for the present study and, if applicable, for the original study on which the present article is based	Page 10.	"This research was supported by a grant of the Korea Health Technology R&D Project through the Korea Health Industry Development Institute (KHIDI), funded by the Ministry of Health & Welfare, Republic of Korea (grant number: RS-2024–00335936)."

STROBE, Strengthening the Reporting of Observational Studies in Epidemiology.
